# Basic and applied biology of the primate reproductive tract – a symposium in honor of the career of Dr Robert M Brenner: Introduction and overview

**DOI:** 10.1186/1477-7827-4-S1-S2

**Published:** 2006-10-09

**Authors:** Robert M Brenner

**Affiliations:** 1 Division of Reproductive Sciences, Oregon National Primate Research Center, Oregon Health & Science University, 505 NW 185th Avenue, Beaverton, OR 97006, USA

## Introduction

In 1962, the National Institutes of Health dedicated the first Primate Center in the United States, located in Beaverton, Oregon and closely associated with what was then called the University of Oregon Medical School, now known as Oregon Health & Science University. Within a few years, the Oregon Regional Primate Research Center was followed by 6 others in various cities across the country that were affiliated with many distinguished universities. In recent years another institution has joined the unique "club" now known as the National Primate Centers. The key aim of these centers was to explore the biology of nonhuman primates with the ultimate goal of improving human health. The original planners at NIH who conceived the Primate Centers deserve great credit, and this symposium is in part a testament to their foresight.

While no animal model is perfect, all agree that nonhuman primates are superbly useful for probing key aspects of human biological systems. Early on it was recognized that the reproductive system in female nonhuman primates was almost identical to that of women. Consequently, the Oregon Center focused its efforts on reproductive physiology. Evidence accumulated that numerous gynecological problems that afflict women such as premature birth, pelvic disorders, dysfunctional bleeding and endometriosis had their counterparts in female nonhuman primates. Consequently, strong collaborations developed between Primate Center scientists and Ob-Gyn physicians, nationally and internationally, to probe the mechanisms underlying these disorders. Many of the papers presented at this symposium provide summaries of such collaborative efforts, and all the reviews provide helpful perspectives and important insights into the field of endometrial physiology, with examples drawn from both human and nonhuman primates.

## Cellular and molecular regulation of the primate endometrium: A perspective

Leading off, Dr. William Okulicz, Ph.D. Associate Professor, Department of Obstetrics and Gynecology, University of Massachusetts Medical School, Worcester, MA, provided an insightful, historical review of our knowledge of the uterus, entitled: "Cellular and molecular regulation of the primate endometrium: A perspective". Beginning with Aristotle's' writings in which the human uterus was erroneously described as bicornuate, through the 15^th ^century when Da Vinci and Vesalius made accurate drawings based on autopsy specimens, Dr. Okulicz summarized the early foundations of uterine biology. Further, he highlighted the work of Allen, Doisy, Corner and Markee in the early 1900's; pioneers whose classic experiments with nonhuman primates revealed that endometrial cycles were controlled by estrogens and progestins. The discovery of a high affinity estradiol receptor by Elwood Jensen's lab, the subsequent development of specific antibodies to many receptors, and the use of immunocytochemistry to localize hormonally sensitive cells in complex tissues were succinctly presented. Finally, Professor Okulicz concluded his historical review with an up to date presentation of how microarray and subtractive hybridization can be used to evaluate cyclic changes in gene expression in the macaque endometrium.

## Application of functional genomics to primate endometrium: Insights into biological processes

Dr. Linda Giudice, M.D, Ph.D., Professor and Chair of the Department of Obstetrics, Gynecology and Reproductive Sciences, University of California, San Francisco, CA, carried the theme of global analysis of gene expression into the realm of human endometrial physiology in her presentation: "Application of functional genomics to primate endometrium: Insights into biological processes". While warning of the difficulties of obtaining "normal" human endometrial specimens, she summarized the recent literature, including her own pioneering work, and suggested that the Noyes histological dating techniques, well known to be highly variable and observer-dependent, can now be complemented and will eventually be replaced by stage-specific patterns of gene expression based on microarray and bioinformatic analysis. She characterized the endometrium as "the anatomic prerequisite of the continuation of the species" and indicated that a full, functional genomic and proteomic analysis of this dynamic tissue is within our grasp. By including a series of very well designed figures and tables, she summarized in pictorial form the regulation of specific genes during each phase of the human endometrial cycle. Moreover, she segregated these genes into metabolic, angiogenic, immune and other functional classes in highly original and useful ways. Researchers who use genomic and proteomic approaches to explore endometrial physiology will be well served by this succinct and brilliant review.

## Regulation of human endometrial function: Mechanisms relevant to uterine bleeding

Hilary O.D. Critchley, Professor of Reproductive Medicine, New Royal Infirmary, and Centre for Reproductive Biology, University of Edinburgh, Edinburgh, Scotland focused on a key phase of the human menstrual cycle, namely, menstruation itself, in her presentation: "Regulation of human endometrial function: Mechanisms relevant to uterine bleeding". She noted that "...disorders of the menstrual process are one of the banes of modern women", and emphasized that an understanding of the normal menstrual process at a molecular level is essential to the development of rational therapies for the bleeding disorders so often seen in the clinic. She identified progesterone withdrawal as the key precipitating factor in the complex cascade of events that result in menstrual breakdown, and she reviewed the role of steroid receptors, steroid metabolizing enzymes, hypoxia inducing factors, matrix metalloproteinases and inflammatory mediators in menstrual induction. She emphasized that menstruation consists of two phases, a set of early events, including vasoconstriction of the spiral arteries, likely to be reversible, and a set of later, nonreversible events involving proteolysis of the extracellular matrix and ultimate sloughing. Her summary included a comprehensive diagram depicting a modern synthesis of each step in the menstrual cascade, from vasoconstriction through proteolytic destruction. Her review made clear that further research on the underlying details of the menstrual cascade will greatly benefit the health of women.

## A critical period of progesterone withdrawal precedes menstruation in macaques

Dr. Ov D. Slayden, Ph.D., Scientist, Division of Reproductive Sciences, Oregon National Primate Research Center, Beaverton, OR, expanded on the theme that menstruation is a two phase process in his presentation entitled: "A critical period of progesterone withdrawal precedes menstruation in macaques". He induced artificial menstrual cycles in ovariectomized macaques by sequential insertion and removal of subcutaneous implants of estradiol (E) and progesterone (P). When P was withdrawn at the end of the cycle, there was approximately a 36 hour time window during (but not after) which, menstruation could be blocked by adding back P. After ~36 h, menstruation became inevitable. Dr. Slayden described the effects of P withdrawal and P add back on the regulation and localization of various matrix metalloproteinases in the premenstrual and menstrual macaque endometrium, and provided a succint summary of the literature on the potential roles of NFκB, its inhibitor, IκB, and the various enzymes involved in regulation of endometrial prostaglandins and cytokines. These latter factors may be the ones that provide the initial anoxic insult that follows P withdrawal. The work provides convincing experimental evidence confirming the view that menstruation is a two-phase process.

## A baboon model of endometriosis: implications for fertility

Dr. Asgi Fasleablas, Ph.D., Professor of Physiology and Director, Center for Women's Health & Reproduction, Department of Obstetrics and Gynecology, University of Illinois, Chicago, Ill, introduced the problem of endometriosis by presenting: " A baboon model of endometriosis: implications for fertility". Although women and nonhuman primates are the only species in which endometriosis occurs naturally, there have been numerous attempts to model this disease through transplantation and other techniques in laboratory rodents. However, the absence of cyclical menstrual breakdown in these animal models limits their value. The experimental animals of choice for study of endometriotic growths are the nonhuman primates, and Dr. Fasleabas' laboratory has developed and used techniques for transplanting the menstrual endometrium into peritoneal sites under laparoscopic control, and for sampling both the eutopic and ectopic tissues for analysis. He reviewed both his findings and the general literature in the field, and noted that matrix metalloproteinases (MMPs), proteolytic enzymes that are usually suppressed in the luteal phase, are upregulated during the secretory phase in both the ectopic endometriotic and the eutopic endometrial samples, indicating crosstalk between the two sites. He emphasized that the high levels of MMPs present in endometrial fragments during retrograde menstruation facilitate implant invasiveness. The role of angiogenic factors, including VEGF and CYR61, in endometriotic growth, and of other factors associated with diminished fertility were critically presented. Finally, Dr. Fasleabas discussed the "chicken vs egg" problem, namely, whether the aberrant properties of the eutopic endometrium are inherent (and came first) or whether they are secondarily induced by the disease. His working hypothesis is that peritoneal endometriotic lesions can induce changes in the eutopic endometrium through communication pathways that are currently unknown and need to be elucidated. Students of endometriosis will benefit in important ways from this scholarly review.

## Role of nonhuman primate models in the discovery and clinical development of selective progesterone receptor modulators (SPRMs)

Dr. Kristof Chwalisz, M.D., Ph.D., Therapeutic Area Head of Women's Health at TAP Pharmaceutical Products, Inc., Chicago, III, addressed new ways to treat human endometriosis in his presentation entitled: "Role of nonhuman primate models in the discovery and clinical development of selective progesterone receptor modulators (SPRMs)". These molecules, in his words, "represent a new class of PR ligands that exert clinically relevant tissue-selective progesterone agonist, antagonist, partial, or mixed agonist/antagonist effects on... progesterone target tissues... ". These remarkable compounds have an equally remarkable history. The field began with the discovery of progestin antagonists, typified by RU 486, at Roussel-UCLAF in 1981. Subsequently, Walter Elger at Jenapharm GmbH (Jena, Germany) discovered a new class of mixed agonist-antagonists, called mesoprogestins because they functioned "midway" between progestins and antiprogestins; these molecules have been renamed SPRMs. Dr. Chwalisz reviewed the clinical studies his group conducted which clearly demonstrated the value of these compounds in the treatment of endometriosis as well as leiomyoma and dysfunctional bleeding. In addition, he summarized preclinical studies in nonhuman primates which showed that SPRMs had endometrial antiproliferative effects. The nonhuman primate studies, he noted "...played a key role in the conceptualization and discovery of tissue selectivity of SPRMs". In concluding, he reviewed the various hypotheses concerning the molecular mechanism of action of these new therapeutic agents and suggested that the tissue selective effects of SPRMs are controlled by tissue differences in steroid-receptor coregulators. New research to explore these aspects of SPRM molecular biology will be both exciting and important.

## Estrogen receptor-alpha (ER-alpha) and defects in uterine receptivity in women

Dr. Bruce A. Lessey, M.D., Ph.D., Director, Division of Reproductive Endocrinology, Center for Women's Medicine, Greenville Hospital Systems, Greenville, South Carolina, continued the emphasis on aberrant endometrial states with his presentation entitled: "Estrogen receptor-alpha (ER-alpha) and defects in uterine receptivity in women". Dr. Lessey provided a novel and interesting hypothesis, namely, that under progesterone influence "...the decline in ER-alpha... in the endometrium... may be a critical event, releasing an inhibitory influence on specific genes and providing a signal for endometrial receptivity to commence". As an example of a critical gene inhibited by estrogen action, Dr. Lessey focused on the alphaV/beta3 integrin, a gene closely associated with successful human implantation. During the normal luteal phase, ER alpha declines and the expression of alphaV/beta3 increases in the luminal epithelium. But in cases of luteal phase defect and endometriosis, ER alpha remains aberrantly high and the alphaV/beta3 integrin gene is not expressed. Further, estrogen can inhibit beta3 expression in Ishikawa cells in culture, and this inhibition was blocked by an antiestrogen, strongly implicating ER alpha as a key mediator of alphaV/beta3 suppression. Dr. Lessey suggested that defects in regulation of ER alpha may be at the root of various defects in uterine receptivity and that... "the presence of ER-alpha in mid-luteal endometrium may be the best biomarker of a dysfunctional endometrium". This novel hypothesis should stimulate a burst of new research in the field of uterine receptivity.

## The role of HLA-G in human pregnancy

Finally, Dr. Joan Hunt, Ph.D., Professor of Anatomy and Cell Biology, University of Kansas Medical Center, Kansas City, Kansas, discussed the ultimate endpoint of endometrial function, namely, establishment and maintenance of pregnancy in her overview : "The role of HLA-G in human pregnancy". She noted that... "The enigma of mammalian pregnancy, which usually succeeds despite genetic differences between the mother and her embryo/fetus, has intrigued immunologists for half a century." It was Sir Peter Medawar who stated that the pregnant uterus is an immune privileged site, and in this review, Dr. Hunt outlined the maternal and fetal interactions which underlie that privileged state. Her main focus was on the molecular biology of the HLA-G group of antigens, their soluble and membrane forms, and the evidence for their role in immune privilege. She pointed out that fetal cytotrophoblasts, as they migrate towards the decidua, serially express different members of the HLA-G family, most likely in response to changes in oxygen tension gradients. She summarized the functional evidence that HLA-G antigens can suppress the immune responses of various hematopoietic cells and concluded that HLA-G molecules are the key players that regulate immune responses in the pregnant uterine environment, though other factors may also play important roles. There are orthologues of the HLA-G molecular family in nonhuman primates, especially the *Paan*-AG gene, which has both similarities to and differences from human HLA-G genes. Dr. Hunt noted that further experimental analysis of the role of the *Paan*-AG gene family in protecting pregnancy in nonhuman primates would greatly improve our understanding of human semiallogeneic pregnancy.

The symposium was also greatly honored by the attendance of Dr. David T. Baird, Professor, Department of Obstetrics and Gynecology, Centre for Reproductive Biology, University of Edinburgh Edinburgh, UK, who contributed immensely to the program by discussing his recent work on ovarian folliculogenesis, which will be published in a separate venue.

In sum, this symposium consisted of a series of brilliant and exciting presentations from world-renowned authorities covering the range of normal and abnormal endometrial function, from menstruation to implantation, from bleeding disorders to endometriosis, and from genomic profiling to the molecular biology of the HLA-G gene. There is much here to intrigue and benefit both the avid student of endocrinology and the busy clinician striving to improve women's health. Enjoy!

